# Standardised outcomes in nephrology – Haemodialysis (SONG-HD): study protocol for establishing a core outcome set in haemodialysis

**DOI:** 10.1186/s13063-015-0895-7

**Published:** 2015-08-19

**Authors:** Allison Tong, Braden Manns, Brenda Hemmelgarn, David C. Wheeler, Peter Tugwell, Wolfgang C. Winkelmayer, Wim van Biesen, Sally Crowe, Peter G. Kerr, Kevan R. Polkinghorne, Kirsten Howard, Carol Pollock, Carmel M. Hawley, David W. Johnson, Stephen P. McDonald, Martin P. Gallagher, Rachel Urquhart-Secord, Jonathan C. Craig, on behalf of the SONG-HD Collaboration

**Affiliations:** Sydney School of Public Health, The University of Sydney, Sydney, Australia; Centre for Kidney Research, The Children’s Hospital at Westmead, Westmead, Sydney Australia; Departments of Medicine and Community Health Sciences, Libin Cardiovascular Institute and O’Brien Institute of Public Health, University of Calgary, Calgary, Canada; Centre for Nephrology, University College London, London, United Kingdom; Department of Medicine, University of Ottawa, Ottawa, Canada; Section of Nephrology, Baylor College of Medicine, Houston, United States; Renal Division, Ghent University Hospital, Ghent, Belgium; Crowe Associates, Ltd, Oxon, United Kingdom; Monash Medical Centre and Monash University, Clayton, Australia; Department of Epidemiology and Preventative Medicine, Monash University, Melbourne, VIC Australia; The Institute for Choice, University of South Australia, Sydney, Australia; Renal Division, Kolling Institute, Sydney, New South Wales Australia; Queesland School of Medicine, University of Queensland at Princess Alexandra Hospital, Brisbane, Australia; Translational Research Institute, Brisbane, Australia; Metro South and Ipswich Nephrology and Transplant Services (MINTS), Brisbane, Australia; Central Northern Adelaide Renal and Transplantation Service, Royal Adelaide Hospital, Adelaide, Australia; Faculty of Health Science, University of Adelaide, Adelaide, Australia; Concord Clinical School, University of Sydney, Sydney, Australia; Renal and Metabolic Division, The George Institute, Sydney, Australia

**Keywords:** Core outcome set, Outcomes research, Patient-centred outcomes clinical trials, Dialysis, Haemodialysis, Chronic kidney disease

## Abstract

**Background:**

Chronic kidney disease is a significant contributor to mortality and morbidity worldwide, and the number of people who require dialysis or transplantation continues to increase. People on dialysis are 15 times more likely to die than the general population. Dialysis is also costly, intrusive, and time-consuming and imposes an enormous burden on patients and their families. This escalating problem has spurred a proliferation of trials in dialysis, yet health and quality of life remain poor. The reasons for this are complex and varied but are attributable in part to problems in the design and reporting of studies, particularly outcome selection. Problems related to outcomes include use of unvalidated surrogates, outcomes of little or no relevance to patients, highly variable outcome selection limiting comparability across studies, and bias in reporting outcomes. The aim of the Standardised Outcomes in Nephrology-Haemodialysis (SONG-HD) study is to establish a core outcome set for haemodialysis trials, to improve the quality of reporting, and the relevance of trials conducted in people on haemodialysis.

**Methods/design:**

SONG-HD is a five-phase project that includes the following: a systematic review to identify outcomes that have been reported in haemodialysis systematic reviews and trials; nominal group technique with patients and caregivers to identify, rank, and describe reasons for their choices; qualitative stakeholder interviews with patients, caregivers, clinicians, researchers, and policy makers to elicit individual values and perspectives on outcomes for haemodialysis trials; a three-round Delphi survey with stakeholder groups to distil and generate a prioritised list of core outcomes; and a consensus workshop to establish a core outcome set for haemodialysis trials.

**Discussion:**

Establishing a core outcome set to be consistently measured and reported in haemodialysis trials will improve the integrity, transparency, usability, and contribution of research relevant to patients requiring haemodialysis; ensure that outcomes of relevance to all stakeholders are consistently reported across trials; and mitigate against outcome reporting bias. Ultimately, patients will be more protected from potential harm, patients and clinicians will be better able to make informed decisions about treatment, and researchers and policy makers will be more able to maximise the value of research to the public

## Background

Chronic kidney disease (CKD) is a leading contributor to mortality and morbidity [1–3]. Globally, the number of people depending on kidney replacement therapy in the form of kidney transplant or dialysis continues to increase [4, 5]. Dialysis consumes a disproportionately high proportion of the health-care budget in many countries [6–9]. Patients on dialysis have a 15 % annual mortality rate, which is 15–100 times higher than that of the general population [10–13]. Most patients receiving kidney replacement therapy receive a form of dialysis, requiring many hours of treatment each week. Patients must also endure treatment burdens associated with polypharmacy, invasive procedures, and difficult diet and fluid restrictions, all of which profoundly disrupt nearly all aspects of life. Studies have consistently shown that patients requiring dialysis report a significant decrement in quality of life, even lower than that reported in patients with metastatic cancer and other chronic diseases [14, 15].

This escalating burden has spurred an increase in randomised trials and other forms of research in the dialysis setting. From 2004 to 2014, 1500 reports of randomised controlled trials (RCTs) (2000 including conference abstracts) in haemodialysis were identified in the Cochrane Renal Group Specialised Register [16]. Yet there has been no substantial improvement in clinical, quality of life, and mortality outcomes for patients on dialysis [17, 18]. The reasons for this are multiple and complex but may be attributed in part to fundamental problems in the design and reporting of these studies, particularly outcome selection. Problems relating to outcomes include highly variable outcome selection, outcome reporting bias, using outcomes of little patient relevance, and the use of unvalidated surrogate outcomes [19–22]. Most trials that have assessed survival have not shown significant improvements in mortality; and studies typically measure biochemical parameters, so it is unclear whether the interventions improve outcomes that are clinically relevant and important to patients.

Clinical trials aim to evaluate whether interventions are safe and efficacious for patients by comparing their relative effects on outcomes chosen by the investigators. The heterogeneity in outcomes measured and reported in trials often renders the combination and comparison of trial results impossible [23]. This creates difficulties in interpreting the observed treatment effect and in making evidence-based health-care decisions. For example, standard approaches to evidence synthesis such as meta-analysis cannot be used because of heterogeneity in trial outcomes.

Outcome reporting bias can also threaten the validity of meta-analysis and reliability of evidence [24, 25]. This occurs when there is selective reporting within studies, at the level of individual outcomes, based upon observed favourable outcomes for the intervention [26]. Also, omitting or inaccurately reporting outcomes, such as adverse events, misleads and misinforms patients and clinicians, and this may result in patient harm.

A preoccupation with mortality may preclude study of outcomes that are relevant to patients in living their life. All researchers would regard mortality as an important outcome, but an emphasis on mortality may detract from other outcomes that impact a patient’s quality of life. This is particularly important in a setting like dialysis where very few interventions have been shown to improve survival. The absence of mortality as a priority outcome in research prioritisation activities undertaken from patients’ perspectives has been observed in cancer, mental health, and pulmonary disease [27, 28]. Two research priority setting partnerships in CKD conducted in Australia and Canada, patients, caregivers, and health professionals participated in facilitated discussion and prioritisation surveys to identify, rank and deliberate on research priorities [29, 30] also found there was minimal discussion about mortality. Outcomes determined to be most relevant to the top 10 haemodialysis research questions included quality of life, satisfaction, anxiety, fatigue, employment, interdialytic weight gain, and capacity for self-management [29]. This strongly suggests that patients focus on living well with CKD rather than dying from it.

Most studies do not report endpoints that are relevant, meaningful, and important for patients, because patients have not been included in planning of the studies. Instead, study investigators from academia and industry typically define the outcomes to be measured in research [22, 31]. Surrogate or composite endpoints based on investigators’ input are frequently selected in trials to reduce the cost, time, and sample size requirements and to facilitate rapid market access of new drugs. There is compelling evidence of the dangers of relying on surrogate endpoints in trials [32]. For example, erythropoietin was approved to treat anaemia in patients with CKD on the basis of improvements in haemoglobin, but targeting normal haemoglobin levels was later found to increase mortality [32, 33] but without a significant increase in quality of life [34].

The lack of patient involvement in a research priority setting in CKD in general and in establishing research outcomes more specifically is problematic given the observed mismatch of priorities between patients and clinicians in CKD [29, 35–39]. For example, patients on dialysis are willing to sacrifice survival for freedom to travel and prioritise caregiver respite [36, 37], outcomes which are not “typically” assessed in research. Patients attach a plethora of different values to outcomes, such as empowerment, control, independence, social acceptance, family, guilt, normality, equity, freedom, flexibility, productivity, and security [36, 40–44]. Patient-centred care involves shared decision making and disease management between a patient and their clinician which take into account the patient’s priorities and values and which ultimately can encourage adherence to treatment and improve patient-centred outcomes [45]. Delivering patient-centred care requires an understanding of outcomes that are important and relevant to patients.

In 2014, the *Lancet* reported that 85 % of the US$240 billion expended on health research in 2010 was wasted because of problems in the design, conduct, analysis, and reporting of research [20]. That article emphasised that “waste is caused when potential users’ needs are ignored” [20] (that is, when researchers do not assess the effects of interventions in terms of functional, social, and emotional well-being or adverse reactions and long-term outcomes). This is important given that much of research is publicly funded and that it is allocated with the ultimate aim to improve the health and well-being of populations.

Globally, there are increasing calls to develop core outcome sets to ensure that trials include outcomes that are relevant and important to stakeholders. The Outcome Measures in Rheumatology (OMERACT) initiative was formed in 1992 to identify and improve relevant health outcome domains in rheumatology through an iterative consensus process involving relevant stakeholder groups, including patients [19, 46, 47]. OMERACT outcomes have been endorsed by the World Health Organization (WHO) and the US Food and Drug Administration and have improved the reporting and relevance of outcomes in rheumatology trials [47, 48]. The OMERACT methodology has been applied successfully in cancer [49], otitis media [50], eczema [51], and chronic pain [52]. In 2010, the international Core Outcome Measures in Effectiveness Trials (COMET) organisation was launched to facilitate the development and application of “core outcome sets” [53, 54]. Core outcome sets represent the minimum that should be measured and reported in all clinical trials for a specific condition; however, they are not meant to be definitive [53]. The intention is that the core outcomes be collected and reported to allow the results of trials and other studies to be compared, contrasted, and combined as appropriate; researchers are at liberty to collect and explore other outcomes. A core outcome set does not exist for CKD.

This project will initially focus on haemodialysis as it is the predominant dialysis treatment modality worldwide. The ultimate aim of SONG is to develop a core outcome set across the treatment spectrum of CKD, including early-stage CKD (non-dialysis-dependent), haemodialysis, peritoneal dialysis, and kidney transplantation. The specific aim of SONG-HD is to establish a core outcome set which is for haemodialysis trials and which will be used in other forms of research. This is particularly important in CKD, where registries of people on dialysis or who have been transplanted are standard practice. To achieve this aim, the following specific objectives will be addressed: (1) to describe the scope, quality, and consistency of outcomes used in haemodialysis trials; (2) to identify outcomes that are important to patients, caregivers, clinicians, and policy makers; (3) to ascertain the attitudes, values, and beliefs underpinning their priorities for outcomes; (4) to generate an evidence-informed, consensus-based prioritised list of core outcome domains; and (5) to establish a core outcome set for haemodialysis trials.

## Methods/design

SONG-HD is a five-phase multi-method project that includes systematic reviews, nominal group technique/focus groups, semi-structured interviews, Delphi surveys with best-worst/choice experiments, and a consensus workshop. Our methodological framework is based on validated processes developed by the OMERACT initiative [19] that is endorsed by the WHO as a “successful approach” for identifying core outcomes [55].

### Phase 1: Systematic review of outcome domains reported in haemodialysis trials

A systematic review will identify and compare outcomes reported in systematic reviews and RCTs of interventions for adults on haemodialysis.

### Search strategy

A comprehensive search will be conducted in the Cochrane Central Register of Controlled Trials without time and language restrictions.

### Types of studies and interventions

Cochrane systematic reviews and RCTs will be included. For feasibility, the sampling frame for RCTs will be limited to RCTs included in published Cochrane systematic reviews as data extraction will be conducted at the trial level. Any intervention used to manage and treat patients on haemodialysis will be included.

### Types of participants

Adult patients (age of at least 18 years) on haemodialysis.

### Exclusion criteria

Studies that exclude patients on haemodialysis.

### Eligibility of studies

Two reviewers will independently assess all records obtained. Full copies of all potentially relevant systematic reviews and RCTs will be assessed independently by the two reviewers, and any disagreement on the eligibility of included studies will be resolved through discussion.

### Assessment of methodological quality

An assessment of methodological quality will be undertaken by using the Harman (2013) [50] appraisal framework which addresses the rationale, selection, and reporting of outcomes. This will be assessed independently by two reviewers.

### Data extraction

From each RCT, details of the author, year of publication, population (that is, only haemodialysis), number and age of participants, and the intervention and comparator will be recorded. From each study, all outcomes will be extracted by two authors. All outcomes will be extracted unless it is specifically stated in the RCT that the outcomes were measured and reported in the non-HD patients only.

### Data analysis and presentation

All eligible systematic reviews and RCTs will be tabulated. The outcomes will be initially classified according to the broad OMERACT categories of (i) mortality, (ii) life impact, (iii) pathophysiological manifestations, (iv) resource use and economic impact, and (v) toxicity, side effects, and adverse events. Within each category, the outcomes will be grouped into more specific outcome domains. These domains will be reviewed by the Executive Committee to assess the appropriateness of the domain name and grouping of outcomes. The frequency of outcomes reported across systematic reviews and RCTs will be assessed, and the scope and consistency of outcome reporting will be evaluated.

### Phase 2: Nominal group technique with patients and caregivers

To ensure that patient-centred outcomes are identified, patients and caregivers will be asked to identify and rank outcomes that are considered relevant and important to them and to discuss reasons for their choices. A combined focus group/nominal group technique will ensure that the outcome domains important to the patients and caregivers be grounded in their own accounts of what matters to them about haemodialysis. The nominal group technique is a transparent process of consensus development based on structured group discussion [27, 56, 57]. This technique is useful for generating ideas and priorities, prevents dominant participants from controlling the discussion, and allows participants the opportunity to raise views and suggestions without direct criticism or rejection of other participants [56].

### Participants and recruitment

Patients and caregivers who have direct experience with haemodialysis will be invited to participate. Approximately 12 nominal groups (involving 8–12 participants, total *n* = 144) will be convened, though the final number of groups will depend on when theoretical saturation, defined as the point when few or no new outcomes or issues are emerging, is reached. Participants will be recruited from participating centres in Australia and Canada in the first instance and purposively sampled to achieve maximum diversity of demographic (age, gender, socioeconomic status, ethnicity, location, and educational attainment) and clinical characteristics (in-centre versus home haemodialysis, diagnosis, duration on dialysis, wait-listing status, and comorbidities) to capture a breadth of opinion. Informed consent will be obtained from all participants.

### Data collection

The nominal groups will be 2 hours in duration and convened in a centrally located venue external to the hospital to minimise the possibility of participants feeling disempowered. All discussions will be audiotaped and transcribed verbatim, and a researcher will record the contextual details around the discussion. The question guide will be developed from the guide we designed to successfully elicit outcomes in kidney transplant recipients [38] and includes the following:

i. Welcome and introduction (5 min)
ii. Focus group discussion (45 min) – Participants will deliberate and discuss experiences of HD and perceived benefits, harms, and complications of haemodialysis and haemodialysis-related interventions.
iii. Nominal group technique (70 min) – Participants will individually identify outcomes they believe are important and relevant. These outcomes will be written on the flipchart and then augmented with outcomes identified from the systematic review (phase 1) and in previous nominal groups. The list of outcomes will be discussed and clarified. A copy of the consolidated list will be printed for participants to individually rank all of the outcomes in order of perceived importance, from 1 (most important) to X (least important). Similarities and differences in ranking will be discussed among the group.

### Data analysis

Nominal group ranking: The highest ranked outcome for each respondent will be assigned a value of 10 through to the least important, which will be assigned a value of 1. Outcomes not ranked in the top 10 will be assigned a value of zero. A mean priority score for each outcome across all groups will be obtained by summing ranking scores and dividing this by the maximum possible ranking score for that item. The maximum possible ranking score for a given outcome will be calculated by multiplying the number of participants who considered the outcome by 10 (the maximum rank). If all participants who ranked an outcome scored it as the most important, the priority score is 100 %, whereas a score of 0 % means that all participants who ranked that outcome did not score it in the top 10 most important outcomes. Mean priority scores and the number of times an outcome was voted in the top 10 will be calculated for all participants. Mean priority scores will also be calculated separately for demographic and clinical characteristics, and differences in mean scores will be assessed by using analysis of variance.

Qualitative analysis: Using an adapted grounded theory approach, as outlined by Corbin and Strauss [58], the transcript will be imported into HyperRESEARCH and reviewed line by line to identify concepts and themes, compared within and across groups, and developed into a coding scheme. The preliminary themes will be discussed with two other investigators to ensure that the full range and depth of data are captured (investigator triangulation). Through a process of constant comparisons between individuals and groups, analytical themes will be developed. The final themes will reflect the beliefs, values, attitudes, and reasons underpinning the participants’ choices and ranking of outcomes.

### Phase 3: Stakeholder interviews

Semi-structured interviews will be conducted to elicit a range and depth of individual values, beliefs, and attitudes toward outcomes, not to quantify frequency of opinion [44, 59]. Reporting will be based on the Consolidated Criteria for Reporting Qualitative Health Research (COREQ) [60].

### Participants and recruitment

Interviews will be conducted with the following stakeholder groups internationally: (i) patients and caregivers, (ii) health-care providers (nephrologists, surgeons, nurses, psychologists, nurses, social workers, and dietitians), and (iii) representatives from research, funding, policy, industry, and other stakeholder organisations. A minimum of 30 patients and 30 caregivers will be recruited from participating centres and via consumer organisations worldwide (including Australia, Canada, Europe, New Zealand, the UK, and the US). A minimum of 60 health-care providers, policy makers, and health professionals from national and international stakeholder organisations will be identified from the investigator’s networks. Participants will be “purposively” identified to obtain a maximum variation of representation in demographics, clinical characteristics (patients), and professional experience and responsibilities (health-care providers and representatives from stakeholder organisations). Recruitment will continue until theoretical saturation has been achieved in each stakeholder group. Informed consent will be obtained from all participants.

### Data collection

The interview guide will incorporate the results from the systematic review and phase 2. Participants will be asked to reflect and talk about (1) the experiences of living with haemodialysis (patients) or caring for patients on haemodialysis (caregivers/health-care providers), (2) benefits and harms of haemodialysis/haemodialysis-related treatment, (3) outcomes believed to be relevant and important to include in haemodialysis trials and why, and (4) the results obtained in phase 2. Face-to-face interviews will be conducted, but if this is not possible, Skype or telephone interviews will be conducted. Each interview will be 30–45 min in duration, and all interviews will be recorded and transcribed verbatim.

### Data analysis

From the transcripts, a list of outcomes mentioned by the participants will be extracted. Also, grounded theory and thematic analysis (as detailed in phase 2) will be used to analyze and describe the reasons for their priorities, and a comparison will be made across stakeholder groups.

### Phase 4: Delphi consensus survey

An international Delphi survey will be conducted to collect opinions and distil the number of outcomes to a prioritised list. The Delphi method is an iterative consensus technique comprising sequential surveys answered anonymously by a panel of participants with relevant knowledge and expertise and gives equal influence to all who participate [57, 61].

### Participants and recruitment

There is no standard sample size required in Delphi processes, though Delphi studies in core outcome development have reported participant numbers ranging from 13 to 222. For this study, the Delphi panel will include a minimum target sample size of at least 400 respondents with patients/caregivers comprising at least half of the total sample size to ensure a numerical balance between patients/caregivers and health professionals. Thus, the aim is to recruit patients (*n* = 100), caregivers (*n* = 100), clinicians (nephrologists [*n* = 100], vascular surgeons [*n* = 20], and nurses [*n* = 20]), allied health professionals (psychologists, social workers, and dieticians) (*n* = 20), researchers (*n* = 20), and policy makers (*n* = 20), and researchers and policy makers (*n* = 30). To ensure maximum variation in sampling, participants will be recruited by using a similar strategy detailed in phase 3, and approximately one third from each stakeholder group with be recruited from participating countries/regions, including Australia, the US, Canada, the UK, continental Europe, and Asia.

Participants will be recruited through participating hospital/university institutions and patient/consumer organisations. All participants will be asked to register their email on www.songinitiative.org prior to the survey launch. Informed consent will be obtained from all participants.

### Data collection

The list of outcomes will be obtained from phases 1–3. For feasibility, the outcomes will be listed individually but grouped under a relevant domain. The survey will be reviewed by the Executive Committee and piloted. The surveys will be completed online by using a unique identifier based on their name and email address to enable identification of participants completing all three rounds of the Delphi survey. At least three reminders will be sent to participants during the Delphi rounds.

*Round 1*: Participants will be asked to rate each of the outcomes (approximately 30 outcomes) by using the (GRADE) process, which recommends a nine-point Likert scale to rank their importance [62]. Rankings of 7–9 indicate outcomes of critical importance, rankings of 4–6 indicate outcomes that are important but not critical, and rankings of 1–3 indicate outcomes of limited importance. All outcomes will be randomised to minimise ordering bias. Participants can suggest additional outcomes and provide reasons for their rankings. The additional outcomes will be re-coded and grouped by two members of the investigator team (AT and RS) and reviewed by the Executive Committee. All outcomes will be carried through to round 2 with the distribution of scores displayed for each outcome (rather than cutoff scores) to avoid masking any major disagreement within the group [61].

*Round 2*: Participants will review the group scores and their own score for each outcome and will re-rank the outcomes (including additional outcomes identified in round 1) by using the nine-point scale and explain reasons for any changes to their scoring.

*Round 3*: Participants will be shown their own score and the distribution of scores for each outcome across all stakeholder groups and for separate stakeholder groups. A summary of the results from phases 2 and 3 will also be provided. Participants will be asked to re-rank all outcomes and to indicate whether they should be included in the core outcomes set. After the GRADE rankings, a number of questions based on a novel priority setting approach using choice experiment methods (for example, best-worst scaling (case 1) [63]) will also be included. Participants will be presented with multiple scenarios that present varying combinations of 4–5 outcomes, developed by using a balanced incomplete block design. They will choose the most important and least important in each scenario. By presenting multiple scenarios to respondents, a full ranking of all attributes, based upon the choices that respondents make, can be estimated.

### Data analysis

For each outcome, the number of participants who scored the outcome and the distribution of scores will be summarised together. Results of the stakeholder group responses will be compared with the whole group response, and the percentage agreement will be used to determine the structure and focus of the consensus conference. Each outcome will be classified according to Table 1. This pre-specified definition is used by OMERACT and other similar initiatives [19, 50, 51] and minimises the chance of consensus being defined post hoc in such a way as to bias the results toward the beliefs of the research team. The choice experiment questions will be analysed initially by using a multinomial logit model for all respondents and also by stakeholder group. More complex model specifications such as a mixed logit model [64], a generalised multinomial logit model [65], or latent class models will also be examined. The overall rankings of outcomes generated from this new method will also be compared to determine its consistency with the widely accepted Delphi approach.

**Table 1:** Delphi consensus definition

Consensus in	Consensus that outcome should be included in the core outcome set	≥70 % participants scoring as 7 to 9 AND <15 % participants scoring as 1 to 3
Consensus out	Consensus that outcome should not be included in the core outcomes set	≥70 % participants scoring as 1 to 3 AND <15 % of participants scoring as 7 to 9
No consensus	Uncertainty about importance of the outcome	Anything else

### Phase 5: Consensus workshop

A face-to-face consensus conference will be held for stakeholders to review, comment on, and endorse the core outcomes set. This conference will be chaired by members of the Executive Committee. Approximately 60 participants will be invited and at least half will be patients/caregivers (*n* = 30). Other participants will include nephrologists, nursing and allied health professionals, researchers, and policy makers. This number is based on the OMERACT consensus workshop. Purposive sampling will be undertaken to ensure maximum variation of demographic and clinical characteristics. Informed consent will be obtained from all participants. All discussions will be audiotaped and transcribed. The conference program is outlined below, though details will depend on the results obtained in phases 2–4.

1. Presentation of results: Detailed results from phases 2, 3, and 4 will be distributed to the participants 2 weeks prior to the conference to allow participants time to reflect and enhance their ability to deliberate and contribution to the discussions. The results will be presented during a plenary session of the consensus workshop, and the outcomes will also be shown according to the consensus classification (Table 1).
2. Breakout group discussion: Participants will be divided into six groups of approximately 10 participants. A trained facilitator will moderate a group discussion on the results from phases 3 and 4, consensus classification of outcomes, similarities and differences across stakeholder groups, and the resolution of any disagreement, uncertainties, or issues identified.
3. Plenary discussion: Each breakout group will present a summary of their discussion. The conference chair will moderate discussion.
4. Endorsement of core outcome set: Participants will be asked to formally endorse (sign off) the core outcomes set, which will include the outcomes classified as “consensus in”.

### Ethics

The Western Sydney Local Health District, Sydney, New South Wales, Australia (HREC2009/6/4.15); Monash Health, Melbourne, Victoria, Australia (13082B); the University of Calgary, Calgary, AB, Canada (REB15-0708); and the University of Sydney (2015/228) provided ethical approval for this study.

## Discussion

The international SONG-HD initiative will generate a core outcome set for haemodialysis trials that is based on the shared priorities of patients, clinicians, researchers, policy makers, and relevant stakeholders. The outcome domains will inform subsequent work in the development of outcome measures (that is, specific tools and thresholds) for evaluating outcomes that are meaningful and relevant to users of the research, who are primarily patients and their clinicians.

The core outcome set will be implemented in systematic reviews and trials via the investigator’s existing links with international and national research and policy organisations. Specifically, this will be achieved by prioritising research that addresses core outcomes and integrating core outcome sets into proposal and protocol templates for trials, guidelines, and systematic reviews. Thus, this project will improve the integrity, transparency, usability, and impact of research relevant to patients requiring haemodialysis; ensure that outcomes of relevance to all stakeholders are consistently reported across trials; and minimise outcome reporting bias. Ultimately, patients will be protected from potential harm, patients and clinicians will be able to make informed decisions about treatment, and researchers and policy makers will be able maximise the value of research to the public.

## Trial status

Recruitment and data collection have commenced.

## Abbreviations

CKD: Chronic kidney disease
OMERACT: Outcome Measures Rheumatoid Arthritis Clinical Trials
RCT: Randomised controlled trial
SONG-HD: Standardised Outcomes in Nephrology-Haemodialysis
WHO: World Health Organization
